# Origin and Classification of Aneurisms

**Published:** 1895-03-30

**Authors:** A. Pearce Gould

**Affiliations:** Senior Assistant Surgeon to the Middlesex Hospital


### The Causation of Aneurisms.

By A. Pearce Gould, F.R.C.S., Senior Assistant Surgeon to the Middlesex Hospital.

II.—ORIGIN (*continued*).

In my former article I discussed the definition of the term aneurism, and in this I wish to refer to the mode in which a "blood tumour communicating with an artery" may arise—the etiology of aneurism. When from any cause a grave alteration occurs in the natural relation between the pressure of the blood within an artery and the resisting power of the arterial wall an aneurism is likely to develop. The causes of aneurism, therefore, at once divide themselves most naturally into two groups—(1) conditions increasing the pressure of the blood in the arteries, (2) conditions weakening the arterial walls. The walls of healthy arteries can resist a force much greater than that to which they are habitually exposed, and from this it follows that changes in the arteries lessening or destroying their power of resistance are the prime factor in most cases of aneurism. The anatomical distinction between the three coats of an artery has long been recognised, and the physiological properties of the different coats have been studied. But it has not been sufficiently insisted on that the inner and middle coats of an artery are very much thicker and stronger than the outer coat, and that this is particularly the case in the largest arteries, which are most often the seat of aneurism. It is just these coats which are affected by disease and injury, and in discussing the causes of aneurism, therefore, the first point to make clear is the part that diseases and injuries play in weakening the inner and middle coats of arteries. Atheroma is primarily a disease of the deeper layers of the inner coat, and when advanced it extends into the middle coat also, and may involve the whole thickness of the tunica intima. Its most constant physical effect is to lessen or even to destroy the elasticity of the vessel. Elasticity is the property that enables a substance to shrink to its former size after being stretched, and it must therefore be distinguished from mere extensibility. Atheroma in some of its stages strengthens an artery in the sense of adding to its power to resist a stretching force. More often it diminishes this power of resistance to stretching, but, however that may be, from first to last it lessens the power of a stretched artery to recoil. The calcification which occurs primarily in the muscle cells of the middle coat rarely leads directly to aneurism, for it converts the vessel into a rigid tube; the primary fatty degeneration of arteries is an affection of the most superficial cells of the inner coat, and its effects are far too trifling to impair the strength or the elasticity of an artery. Syphilitic disease of the inner coat of an artery is now a well-recognised disease, but how far it plays a part in the production of aneurism is open to doubt. In its best known forms it is associated with uneven knobby swelling of the tunica intima, and a marked tendency to occlude the humen of the vessel either by proliferation of the cells of the intima or finally by thrombosis of the blood flowing through a very diseased conduit. The clinical evidence in favour of syphilis as a cause of aneurism is undoubtedly strong; many cases occur in which syphilis is the only apparent cause of an aneurism, and it has been suggested that in these instances when the syphilitic arteritis abates in severity the morbid products are absorbed, and only the injured and enfeebled arterial tissues remain to resist the force of the blood; this condition has not been observed. When an embolus lodges in an artery, a certain amount of inflammation of the vessel-wall ensues, if the embolus is non-infective this inflammation is of low grade, and it only advances to slight softening and weakening of the coats of the vessel, then if the heart's action is vigorous an aneurism is very liable to arise. Injury may weaken an artery in any degree; a slight contusion lacerates the inner coat to a slight extent, a more severe injury ruptures completely the inner and middle coats, and leaves only the thin outer coat to withstand the force of the circulation. A sharp instrument in like manner may partially sever an arterial wall, or it may completely divide it. When an artery is completely severed, the blood, escaping from it may flow out of an external wound, or may infiltrate widely the planes of cellular tissure of the part, or collect in some serous cavity; in either case it does not form a circumscribed tumour, and therefore such cases are not to be regarded as aneurisms at all. But in other cases of wound the blood is confined within definite limits by a strong resisting fascia or the matted soft parts, and then the condition is, by our definition, to be known as an aneurism. A combination of arterial disease and injury is quite common. A diseased artery is more liable to be ruptured—partially or completely—by a given contusion than is a perfectly healthy vessel, owing to the lessened elasticity of the diseased vessel. In this way a calcified artery may be the starting-point of an aneurism, for the brittle middle coat may be snapped by a contusion, and the remaining coats of the vessel then yield before the pressure of the blood. Considerable importance has been attached to the loss of support of arteries resulting from the absorption or destruction of the tissues around them. It is certain that in many contracting pulmonary cavities aneurisms are found springing from the arteries running in the walls of the cavities, and projecting into these cavities, Aneurisms are by no means constant under such circumstances, but they undoubtedly occur, and it is very important to notice that they are peculiarly prone to occur in contracting cavities. Negative pressure on the outer side of the artery in these cases may have much to do with their production, but certainly in some cases, if not in all, disease of the inner coat of the artery is found to precede any bulging of the vessel, To sum up, then, we find that the primary factor in the causation of a great majority of aneurisms is some change in the arterial wall lessening its strength and elasticity, and its power of resisting the blood-pressure. This change is most often the result of atheroma, but may arise from syphilitic disease of the artery, from simple (non-infective) embolic arteritis, from contusion, laceration, and wound of an artery, and from loss of external support of an artery. The influence of the blood-pressure in leading to aneurism, and of the various ways in which this pressure is increased, together with the direct and indirect effects of such increase, will form the subject of the next paper.

